# Utilization, Cost, and Affordability of Antihypertensive Therapy in Bulgaria

**DOI:** 10.22037/ijpr.2020.113660.14418

**Published:** 2021

**Authors:** Zornitsa Mitkova, Guenka Petrova

**Affiliations:** *Department of Organization and Economy of Pharmacy, Faculty of Pharmacy,Medical University of Sofia, Sofia, Bulgaria.*

**Keywords:** Antihypertensive medicines, Bulgaria, Cost analysis, Affordability, Utilization

## Abstract

ACE- inhibitors, angiotensin receptor blockers, beta-blockers, Ca- antagonists are recommended as first-line monotherapy for hypertension. The aim of the current study is to analyze expenditures paid by the National Health Insurance Fund (NHIF) after introducing the budget cap cost-containment measure and its impact on affordability and utilization. The study is a retrospective, observational analysis of expenditure on main groups’ antihypertensive medicines: beta blockers, calcium channel blockers, ACE- inhibitors, and AT receptor blockers. The cost paid by the NHIF two years before (2016-2017), and after (2018-2019) the introduction of the budget cap measure was evaluated. Utilization and affordability data covering antihypertensive therapy were retrospectively calculated and analyzed during 2016-2019. The reimbursed expenditures on sartans, ACE-inhibitors, and β-blockers decreased in absolute terms in 2019 compared to that in 2016. There are no statistically significant differences, excluding the group of sartans. The result reveals decreasing utilization of ACE-inhibitors and β-blockers, which is the most significant for enalapril and bisoprolol. Affordability increases during the observed period because less than a working day income is sufficient for monthly therapy. Patients with hypertension in Bulgaria have access to affordable first-line antihypertensive medicines. Despite the stable and low prices, utilization mainly decreases. The reimbursed amount is reduced with a low rate or remains similar to that found at the beginning of the observed period. The results of the implemented budget cap as a measure to control NHIF cost are not evident and not fully expressed on the market for the first-line antihypertensive therapy.

## Introduction

Hypertension is one of the most significant risk factors and the leading cause of ischaemic stroke, intracranial haemorrhage, kidney disease, disability, and high mortality (1-4). WHO data reveal that systolic blood pressure in many European countries decreases last decade with the most significant rate in Northern and Western European countries. Thehighest prevalence of hypertension is found in Estonia and Moldova, whereas it is at least common in the UK, Israel, and Norway, among both EU and outside EU countries (5). The mean systolic blood pressure ranged from 135 mmHg to 134.1mmHg in males, whereas from 135.5 to 125.3 among femalesduring 1980 and 2014 in Bulgaria (6).

Early control of hypertension reduces the risk of heart disease, peripheral vascular disease, and the associated costs for complications (1, 7).Overall expenditures for CVD are estimated about€210 billion annually in Europe. 53% of themdue to direct health care costs, 26% to productivity losses, and 21% to the informal care of people with CVD (6). The proportion of CVD expenditure as part of total healthcare expenditure is from 10% in Sweden to more than 22% in Bulgaria. Share of CVD costs remain significantly high, despite its declining latest years (8).

Hypertension is among the most prevalent chronic conditions, which along with diabetes and hyperlipidaemia lead to the highest avoidable healthcare costs (9). ACE inhibitors,angiotensin receptor blockers (ARBs), beta-blockers, Ca- antagonists, and diuretics have successfully reduced blood pressure and cardiovascular events in randomized clinical trials, making them the first-line recommended therapy for hypertension (10). Antihypertensive medicine prescriptions rise significantly between 2000 and 2013. A systematic review led to the conclusion that the proportions of awareness (32.3% and 37.9%) and treatment (24.9% and 29.0%) slightly increased, while the proportion of hypertension control decreased (8.4% *vs.* 7.7%) during the period 2000 - 2010 (11). Despite rising medicine utilization, only a half of the population with high blood pressure is adequately controlled (12, 13).

Lots of medicines are still unaffordable to patients due to high prices, which makes them too expensive for the lowest-paid workers (14-16). Chronic diseases therapy requires lifelong steady treatment and a combination of two or more medicinal products. It grows up the costs, reduces affordability and medication adherence (17, 18). The direct cost of hypertension is estimated at about 51.3 billion euro by probabilistic prevalence-based decision tree model. The comparison includes Italy, Germany, Spain, France, and England’s payer’s perspective for 10 years. Increasing adherence to therapy to 70% leads to saving about euro 332 million (19).

Payment institutions introduced different types of managed entry agreements termed risk-sharing agreements to control increasing health care expenditure and reduce the financial burden on prescription medicines (20). It is a mandatory condition applied for new or all reimbursed medicines based on agreements between pharmaceutical companies and health insurance funds. Some of the measures focus on the prescribing, other on prices, and the third part on the whole market (21). Budget cap on therapeutic groups expenditures is one of the measures focusing on the whole market. If companies exceedthe budget cap, they have to return the difference. Every cost-containment measure creates different incentives and could influence the reimbursed cost and utilization and affordability of medicines (22).

Two years ago, the national health insurance fund (NHIF) of Bulgaria introduced a budget cap cost-containment measure by separating all reimbursed medicines in 3 groups (group A – medicines for home treatment prescribed after specialists committee recommendations; group B – all other medicines out of group A; and group C – oncology and life-saving medicines) (23).Cardiovascular medicines are part of group B to which most of the commonly and widely used medicines belong. They are prescribed by general practitioners and should be available and affordable to all that need them. 

This regulatory change provokes our interest to explore the budget cap measure effect on the reimbursed expenditures, utilization, price differences, and affordability of mostoften used cardiovascular medicines from the therapeutic groups of beta-blockers, calcium channelblockers, ACE- inhibitors,and AT - receptor blockers. 

For treatment of low-risk uncomplicated hypertension grade 1 patients, as well as hypertension and coronary artery disease or frail older patients, ESC Guidelines and National consensus recommended as first-line monotherapy one of the followed group medicines: ACE-inhibitors, ARB (sartans), CCB, diuretic or beta-blocker, followed by their combinations, as a second treatment step (except combination between two ARBs).Beta-blockers should be considered if there is a specific indication for their use (24, 25). 

The first part of the work presents the analysis of the expenditures paid by the NHIF two years before and after the introduction of the new cost-containment measure (2016-2017 and 2018-2019). The second part presents the changes in utilization of the medicines under consideration, measured in reference DDD/1000inh/day for the same period. The third part focused on the changes in affordability by comparing the working hours needed to pay for a monthly therapy. 

## Experimental

*Design of the study*

The study is a retrospective, observational, macroeconomic analysis of NHIF expenditure on main groups’ antihypertensive medicines: beta-blockers, calcium channelblockers, ACE- inhibitors, and AT receptor blockers, belonging to ATCC07A, C09A, C08C, and C09C from Anatomical Therapeutic Chemical (ATC) classification system. Utilization and affordability data covering antihypertensive therapy were retrospectively calculated and analyzed. The study covers only the medicines reimbursed by the National Health Insurance Fund (NHIF) in Bulgaria during 2016-2019. In total,26 International Nonproprietary Names (INNs) were included in the analysis.

All costs are presented in United States dollars (USD, $) based on the exchange rates of June 2020 (26) 1BGN=0.58 USD

*Data source*

Data for the reimbursed expenditures was collected from the NHIF official register (27). The reimbursed cost was compared during 2016-2019, and it was also used as a basis for calculating medicines utilization from an NHIF perspective.

The reference price per DDD was gathered from the National Council on Prices and reimbursement (NCPR) registers (28). Reference price per DDD is the lowest price per DDD out of all medicinal products under the same INN. The reimbursed value paid by NHIF covers fully or partly only the reference price per DDD. The established reference rate is 25%; 50%; 75% or 100%, depending on the type of disease (29).

The annual data for a number of inhabitants were selected from National Statistical Institute (NSI) database (30). Total number of populations is amended as follow: 7101859 in 2016; 7050034 in 2017; 7000039 in 2018; 6951482 in 2019. The average monthly wage in Bulgaria was also extracted from the NSI database (31): 586.96 USD (2016), 651.34 USD (2017), 679.18 USD (2018), and 761.34USD (2019).

*Cost analysis*

The total reimbursed expenditures and cost due to each INN are retrospectively gathered, summed via Excel 2010 and compared in absolute terms to evaluate their rising or declining during 2016-2019.The cost paid by the NHIF two years before (2016-2017), and after (2018-2019) the introduction of the budget cap measure was extracted for each INN.

The changes in overall reimbursed cost per ATC group between 2016- 2017, 2017- 2018, 2018 - 2019, 2016- 2019, and 2016-2017 versus 2018-2019 were tested with t-test via Excel 2010. *P*-values less than 0.05 are determined as a statistically significant difference.

*Medicines utilisation analysis*

Medicines utilization per INN was evaluated during the observed period. The World Health Organization (WHO) original formula for DDD calculation (32) was modified in order to evaluate utilized reference DDD per 1000 inhabitants daily from NHIF point of view:

Reference DDD/1000 inhabitants/day = ((Reimbursement cost per INN/reimbursement rate)/Reference price per DDD))/365 × number of inhabitants) × 1000

The ratio of reimbursed costs for each INN and reimbursement rate reveals total value paid by NHIF. When dividing by the reference price per DDD, we can calculate how much DDD was sold on the market during the observed year. Finally, we determine the number of utilized reference DDD per 1000 inhabitants annually, using the total population in Bulgaria per year during 2016 -2019.

*Affordability analysis*

Affordability is determined bythe number of working hours per month needed for a patient to purchase monthly therapy medicines. The study assumes that one package of the medicinal product is used for monthly therapy. The lowest and highest price per DDD per INN are calculated among all reimbursed trademarks, depending on package size and dosage form.A monthly therapy cost and average income per hour (if patient work 8 h daily, 22 days in a month) were included in the analysis (33). The methodology for affordability analysis proposed by the World Health Organization was arranged (15) by applying the following steps:

1) Cost of monthly therapy = Lowest or highest price of INN per DDD X 30

2) Wage per hour = ((Average monthly wage)/(22 working days)/(8 working h))

3) Hours wage needed for a monthly therapy =Cost of monthly therapy/Wage per hour

The study considered the treatment as affordable if it costs one-day income or less.

## Results

*Cost analysis*

The reimbursed expenditures decreased in absolute terms in 2019 compared to that in 2016 for sartans, ACE-inhibitors, and β-blockers. It is more evident in the group of β-blockers, where the decline is almost 500,000 USD (Table 1). The NHIF spending on Ca-antagonists has slightly increased.

In the group of ACE inhibitors, the largest share of all reimbursed costs has been paid for lisinopril, enalapril during 2016 and 2017, and for zofenopril and lisinorpil in 2018 and 2019. The highest NHIF expenditure covers valsartan and telmisartan in the group of sartans. The spending on nebivolol is almost half of total b-blockers market, while that of lercanidipine reaches 60 to 70% from all medicines within a group. 

T-test has been performed (Table 1) to test statistically significant differences between reimbursed spending on INNs within a group compared with the same INNs the following years. The difference between the reimbursement amounts paid each year with those paid through the next year has been tested. The results reveal no statistically significant differences between NHIF expenditure over the period 2016 - 2019 for most of the INNs (*p *> 0.05). The exception is the only group of sartans during 2016- 2017 (*p* = 0.03), whereas for the group of ACE inhibitors, the cost difference during 2017- 2018 is near to statistically significant as the *p*-value is 0.054. Statistically significant differences between overall costs during 2016- 2017 versus 2018 - 2019 for each therapeutic group (*p *> 0.05) were not found. Comparing total expenditure two years before and after implementing the budget cap reveals that overall cost declines from 28688869 USD to 26442832 USD. The most obvious difference is found in the group of β-blockers, where the total expenditure for 2018 and 2019 declines almost a million USD compared to that in 2016 and 2017.

The results reveal that total NHIF spending on antihypertensive medicines decreased initially, but later rose in 2019 compared to 2018. Hence, we can’t confirm with certainty the result of implemented measure by the Bulgarian reimbursement institution.

*Utilization in* *reference* *DDD/1000 inh/day*

The calculated utilization of medicinal products from NHIF perspective varies during 2016-2019 (Table 2).

The utilization of ACE inhibitors and β-blockers is decreasing. The decrease in the enalapril (17.008- 12.124 reference DDD/1000 inhabitants/day) and bisoprolol (29.263 - 23.895 reference DDD/1000 inhabitants/day) consumption is the most significant. Total sartans utilization increases with a low rate (from 29.823 to 34.68reference DDD/1000 inhabitants/dayin 2016 and 2019 respectively), while those of Ca-antagonists increased in 2017 compared to 2016, but following decreasing leads to similar value in 2016 and 2019.

*Affordability analysis*

**Figure 1:**
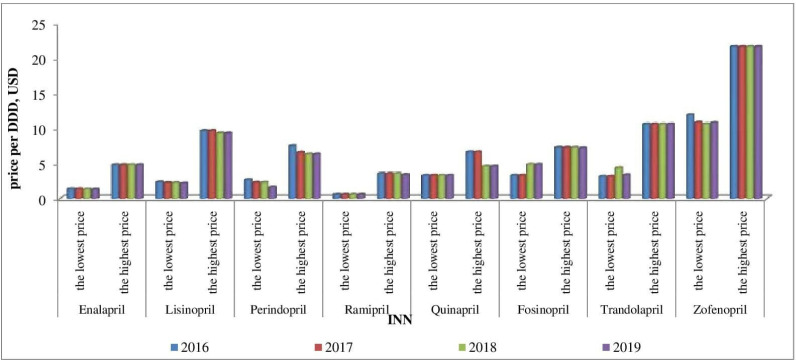
The lowest and the highest ACE-inhibitors prices during 2016-2019

**Figure 2.**
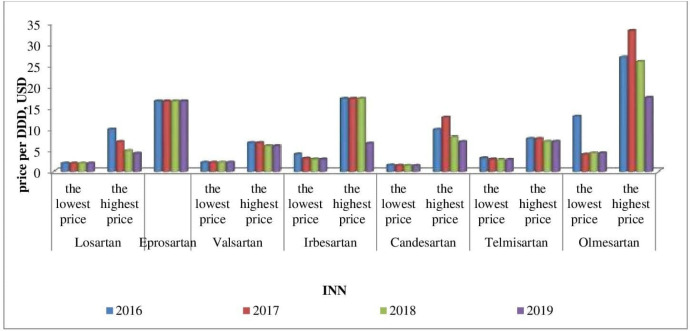
The lowest and the highest sartansprices during 2016-2019

**Figure 3:**
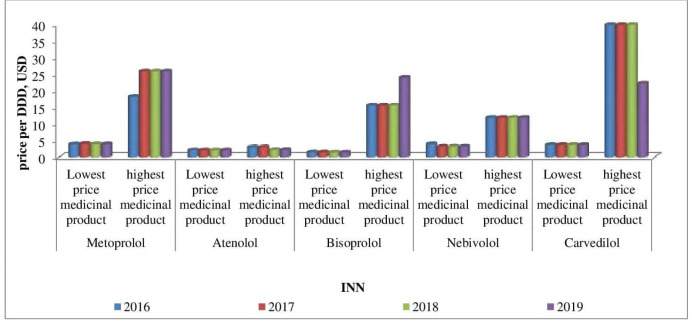
The lowest and the highest β-blockers prices during 2016-2019

**Figure 4:**
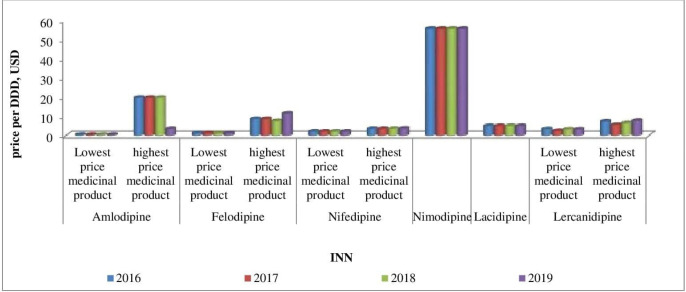
The lowest and the highest Ca-antagonists prices during 2016-2019

Affordability analysis presents the relationship between treatment costs per month and patients’ income. Medicines prices remain stable during 2016-2019 (Figures 1-4). Then affordability changes are due primarily to annual average wage differences.

The number of working hours needed for payment of monthly therapy is calculated based on the lowest or highest priced INNs for treatment with a package (Table 3).

Results show that affordability to therapy increases during the observed period. Indeed, the most expensive INNs available on the Bulgarian market are affordable for monthly therapy. We found as unaffordable 3 medicinal products in 2016, whereas for 2019 is only one (nimodipine). One trade mark nimodipine is available on the Bulgarian market with 16.685 and 14.419 needed working hours for a monthly therapy in 2016 and 2019, respectively.

The most affordable medicinal products are the lowest priced amlodipine (0.141 and 0.121 working hours are needed for a monthly therapy in 2016 and 2019) and ramipril (0.172 and 0.132 working hours are needed for a monthly therapy in 2016 and 2019, respectively).

## Discussion

In Bulgaria National Council on Pricing and Reimbursement approves medicines’ prices. Manufacturer price calculation is based on external(international) referencepricing. The established medicines prices cannot be higher than the lowest manufacturer price in the reference countries. On the other side, for all reimbursed medicines containing the same INN, the reimbursement value is defined at the level of the lowest priced product determined by the value per DDD (defined daily dose). Reimbursement level depends on thetypeof treatment, and typeofthedisease and varies within the scope 25%-100%. Reimbursed medicines are selected into 3 Annexes of the Positive Drug List according to the payment institution. Annex I include outpatients’ medicines paidbythe National Health Insurance Fund. Medicinal products listed in Annex II and used for hospital treatment are covered fromthehospitalbudget. Annex III includes medicines paid by the budget of the Ministry of Health and used for socially significant diseases (AIDS, infectious diseases, vaccines *etc*.) (29).

Our findings reveal decreasing utilizationof antihypertensive medicines over a 4-year period in Bulgaria, which finally resulted in lower NHIF expenditure on reimbursed medicines. Overall drug spending depends on both utilization and unit cost trend and it was driven from rises in the average unit cost and number of prescriptions(34). Overall almost 30% increase in antihypertensive medicines utilization is reported in Germany over a 10-year period.

The highest utilization of ACE inhibitors (55.91 and 50.83references DDD/1000 inh/day) and B-blockers (54.29 and 48.14 DDD/1000 inh/day in 2018 and 2019) from NHIF perspective correspond with the therapeutic guidelines’ recommendation for both therapeutic groups as the first choice of therapy. 

Comparison of cardiovascular medicines utilization in 7 countries (Baltic countries) also shows a rising trend in 2003 and 2012. B-blockers utilization differs from 70,5 to 70,2 DDD/TID in Finland, Ca-antagonists from 42,7 to 85,2 DDD/TID in Denmark, whereas ACE inhibitors reveals the highest level in Lithuania (from 66 to 89,2 DDD/TID) and Finland (from 86,3 to 103,6 DDD/TID) (35).

Similar to our findings have been reported in other studies. In Lithuania utilization of valsartan, amlodipine, and ramipril, followed by enalapril was the highest during 2003 and 2012 (36). We found a significant reduction in enalapril utilization, which hasalso been reported in Lithuania and Germany (37).Consumption of previous market leader enalapril was probably affected by treatment approaches discussing ramipril uses to form the group of ACE inhibitors (38).

The utilization level of amlodipine remain considerable during 2016-2019, presumably based on general recommendations for Ca-antagonists use in hypertension(39,40).Previous study in Germany reported that the widespread use of Ca-antagonists declines within a 10-year period (1998 -2008). There is also observed increased amlodipine consumption (from 5% to 13%) and decreased nifedipine consumption (from 10% to 0.5%). 

The selective b-blockers are currently recommended for the treatment of hypertension (41, 42).Overall consumption of selective b-blockers (and carvedilol) under consideration in our study reveal significant utilization. The results in Sweden and Germany reported that b-blockers are among the most often used for hypertension (43). 

Our findings reveal that sartans (angiotensin II receptor blockers) utilization in Bulgaria is among the lowest, although it confirmed excellent safety and tolerability profile (44, 45).In contrast with our results, comparing CVM utilization in the Slovak Republic and Czech Republic in 2014 shows the highest rate of agents acting on the renin - angiotensin system, followed by Ca -antagonists and beta blockers (46).Further studies are needed to confirm this trend and to explore results in detail. We might assume that this results from the therapeutic competition and patients’ switch to other therapeutic alternatives after batches of valsartan being recalled from the market in 2018 (47).

In general, we can assume that the differences in the utilization rate of antihypertensive medicines during 2016-2019 are mainly due to international guidelines recommendation and rising therapeutic competition.

High medicines prices and out of pocket payment in most countries make them unaffordable for treatment(48,49).Many studies indicate the high cost of drugs and co-payment or family incomes, multiple daily doses, and adverse medication effects as the main factors affecting adherence in patients with chronic diseases (50-54). Finally, it leads to worsening clinical results and insufficient disease control.

CVM is not affordable in most low-income countries (55). Affordability is considered as a dynamic concept depending on CVD therapeutic subgroups, insurance coverage, patients’ characteristics, and medical conditions. From the group of CVM as non-affordable are found antihypertensive and anti-arrhythmic, whereas antihyperlipidemic are the most affordable medicines (56). Most studies included in the systematic review reported average monthly treatment costs for stroke and CHD between $300 and $1000 and monthly costs for hypertension treatment around $22 (57). We consider that CV medicines in Bulgaria are affordable in terms of working hours needed to pay a monthly therapy because less than a day income covers monthly treatment by a package. The needed working hours vary widely from 0.141 for the most affordable amlodipine to 11.929 for the least affordable carvedilol. This fact could encourage patients‘ adherence, and it may alsoimprove clinical results and diseases control in Bulgaria. A study in Iran shows similar results (58). A less than a single day’s wage could be enough for monthly treatment with the lowest-priced generic of the surveyed cardiovascular medicines. The findings reveal both the availability and affordability of medicines for the low-paid unskilled government workers.

A study in Republic of Moldova reveals 1.85 working days in 2006 and 0.84 in 2013 for lowest income worker to purchase 1 month of cardiovascular disease treatment. Introduction of mandatory health insurance and raising household incomes resulted in improved affordability (59).

The study in Portugal reveals that medicines consumption increased by approximately 50% from 2004 to 2012, whereas expenditure decreased(60). It results from frequent use of generics, preferential use of essential medicines, and more rational use of fixed-dose combinations. We also found that expenditures were decreasing in 2018, whereas in 2019, the results are not so homogenous. The price revision showed stable or decreasing prices, which is mainly affected by reference price changes within a group.

Countries in Europe implemented different approaches to control the increasing pharmaceutical costs. EU countries report-ed that setting a budget or expenditure cap is a commonly used approach. Ten countries have implemented a cap on pharmaceutical spending. The pharmaceutical companies are required to pay rebates to public payers if they upper a limit on spending. The budget for public pharmaceutical expenditure and spending cap has been introduced in eight countries (61).

In general, implemented measures in Bulgaria are focused on increasing medicines costs or rising health institution expenditure. External reference pricing directly controls medicines prices, whereas confidential obligatory discounts for all costly medicines between the pharmaceutical company and healthcare payer, price-volume agreement, coverage with evidence development, and the budget cap for all reimbursed medicines are focused mainly on NHIF expenditure. Implemented budget cap for all medicines included in Positive Drug List, Annex 1 is measure guarantying cost predictability and sustainability of NHIF budget. The maximum budget for every group (group A, B, and C) is negotiatedwith the marketing authorization holders. If the budget exceeds the negotiated value companies are paying back the respective proportion of the raise that everyone was causing. 

Despite different cost-containment measures implemented in Bulgaria untill 2018, probably a new approach is needed for guarantying NHIF budget sustainability. At the international level it calls into question the effectiveness of the used tools and confirms that they should be used to align with existing or additional incentives for rational use of medicines (62). Decreasing utilization rate is probably affected by therapeutic competition or increasing FDC utilization in Bulgaria (63). It is not influenced by increasing affordability and stable medicines prices. There is no relationship between levels of medicine consumption and budget cap measure as a factor modifying reimbursement values as they change differently. Further studies are needed to confirm the impact of expenditure cap on NHIF spending on major groups’ medicinal products in the long term and its influence on affordability and utilization. 

Our study has some limitations. First of all, we analyzed medicines utilization from NHIF perspective only for reimbursed medicinal products, and mono products. The estimated utilization reveals consumed reference DDD/1000 inh/day reimbursedby the payment institution. In our study we can‘t precise the number of prescriptions on prevention or treatment of diseases, and the number of patients who consumed two or more medicinal products. The impact of factors as companies’ policies, marketing approaches, and market environment, which influenced medicines utilization and reimbursement, is not considered in that study as there is limited published data.

**Table 1:** Reimbursed spending (USD) on ACE-inhibitors, sartans, β-blockers, Ca-antagonists paid by NHIF and t-test results during 2016-2019

**ACE- inhibitors**				
**Reimbursed spending**				
**INN**	**2016**	**2017**	**2018**	**2019**
Enalapril	511,405.71	540,328.88	448,493.46	343,447.45
Lisinopril	590,146.38	651,343.77	573,730.26	503,153.50
Perindopril	233,942.84	248,805.78	216,521.14	182,688.55
Ramipril	238,757.40	268,053.73	255,466.46	238,124.71
Quinapril	27,008.69	27,639.08	22,831.31	11,400.86
Fosinopril	82,202.70	90,875.29	86,654.02	106,649.54
Trandolapril	36,088.27	34,327.91	27,769.84	52,136.19
Zofenopril	325,397.78	459,367.76	457,440.19	405,124.06
Total amount, USD	2,044,949.77	2,320,742.21	2,088,906.70	1,842,724.84
T-test				
Compared value in	2016 *vs.* 2017	2017 *vs.* 2018	2018 *vs.* 2019	2019 *vs.* 2016
*p*-value	0,066	0,054	0,090	0,379
**AT -receptor blockers, sartans**				
**Reimbursed spending**				
INN	2016	2017	2018	2019
Losartan	129,519.79	150,232.59	132,633.08	121,011.84
Eprosartan	6,648.74	45,570.92	65,228.25	60,530.34
Valsartan	660,202.41	927,478.05	753,880.50	487,833.06
Irbesartan	228,858.58	277,484.86	291,218.88	253,871.38
Candesartan	84,783.38	165,197.02	181,377.33	207,680.99
Telmisartan	637,789.70	691,354.52	599,970.03	427,699.31
Olmesartan	192,228.76	488,473.92	347,809.53	220,038.05
Total	1,940,031.36	2,745,791.89	2,372,117.60	1,778,664.98
T-test				
Compared value in	2016 *vs. *2017	2017 *vs. *2018	2018 *vs. *2019	2019 *vs. *2016
*p*-value	0,038	0,132	0,080	0,635
**β-blockers**				
**Reimbursed spending**				
INN	2016	2017	2018	2019
Metoprolol	1,236,887.09	1,271,196.50	1,177,386.76	1,084,886.17
Atenolol	74,198.94	67,726.22	31,385.28	32,559.58
Bisoprolol	1,899,101.00	1,506,236.16	1,532,821.64	1,441,727.55
Nebivolol	2,509,938.34	2,668,997.56	2,303,477.23	2,716,685.40
Carvedilol	377,999.95	360,887.91	318,151.26	286,310.68
Total	6,098,125.32	5,875,044.36	5,363,222.18	5,562,169.38
T-test				
Compared value in	2016 *vs. *2017	2017 *vs. *2018	2018 *vs. *2019	2019 *vs. *2016
*p*-value	0,654	0,209	0,697	0,372
**Ca-antagonists**				
**Reimbursed spending**				
INN	2016	2017	2018	2019
Amlodipine	464,205.09	491,433.77	455,844.96	404,614.40
Felodipine	203,371.50	183,570.90	148,763.92	126,714.46
Nifedipine	287,756.59	258,034.05	236,510.02	218,079.21
Nimodipine	6,281.45	5,805.65	5,026.62	4,572.37
Lacidipine	340,085.87	350,826.64	335,443.49	309,217.48
Lercanidipine	2,370,073.26	2,702,740.02	2,325,430.60	2,864,809.67
Total	3,671,773.76	3,992,411.02	3,507,019.61	3,928,007.59
T-test				
Compared value in	2016 *vs. *2017	2017 *vs. *2018	2018 *vs. *2019	2019 *vs. *2016
*p*-value	0,387	0,232	0,489	0,659

**Table 2:** ACE-inhibitors, b-blockers, Ca-antagonists, and sartans utilization in reference DDD/1000 inh/dayduring 2016–2019

**INN**	**2016**	**2017**	**2018**	**2019**		**2016**	**2017**	**2018**	**2019**
**ACE inhibitors**					**AT -receptor blockers**					
Enalapril	17.008	18.102	15.722	12.124	Losartan	0.504	3.551	3.157	2.901
Lisinopril	12.078	13.567	12.036	11.103	Eprosartan	0.019	0.129	0.185	0.173
Perindopril	4.253	4.557	3.994	4.250	Valsartan	13.996	19.807	16.214	10.565
Ramipril	19.249	21.770	20.896	19.613	Irbesartan	2.587	4.237	4.798	4.212
Quinapril	0.380	0.391	0.326	0.164	Candesartan	2.626	5.405	6.134	7.072
Fosinopril	1.150	1.280	0.838	1.039	Telmisartan	9.408	11.310	10.271	7.373
Trandolapril	0.528	0.506	0.300	0.731	Olmesartan	0.683	5.643	3.750	2.389
Zofenopril	1.266	1.980	2.028	1.779	Total	29.823	50.082	44.509	34.685
Total	55.912	62.153	56.140	50.803	Ca-antagonists					
Β- blockers					Amlodipine	23.016	24.545	22.930	20.495
Metoprolol	7.092	7.342	7.062	6.553	Felodipine	3.591	3.266	2.665	2.286
Atenolol	0.809	0.744	0.347	0.363	Nifedipine	3.106	2.846	2.627	2.439
Bisoprolol	29.263	23.380	25.228	23.895	Nimodipine	0.003	0.002	0.002	0.002
Nebivolol	14.752	19.473	16.926	15.491	Lacidipine	1.564	1.625	1.565	1.453
Carvedilol	2.372	2.281	2.025	1.835	Lercanidipine	16.018	25.747	16.694	20.710
Total	54.288	53.22	51.588	48.137	Total	47.298	58.031	46.483	47.385

**Table 3:** Working hours needed for monthly therapy

**Working hours needed to cover the low and-high cost therapy**													
**ACE-inhibitors**								**Sartans**					
**INN**		**2016**		**2017**		**2018**	**2019**	**INN**		**2016**	**2017**	**2018**	**2019**
Enalapril	Lowest price medicinal product	0.417		0.376		0.347	0.309	Losartan	Lowest price medicinal product	0.591	0.533	0.511	0.455
Enalapril	highest price medicinal product	1.440		1.298		1.244	1.109	Losartan	highest price medicinal product	2.988	1.901	1.280	0.987
Lisinopril	Lowest price medicinal product	0.699		0.605		0.580	0.495	Eprosartan*		4.957	4.467	4.283	3.820
Lisinopril	highest price medicinal product	2.887		2.602		2.407	2.147	Valsartan	Lowest price medicinal product	0.654	0.589	0.566	0.505
Perindopril	Lowest price medicinal product	0.790		0.616		0.589	0.375	Valsartan	highest price medicinal product	2.030	1.829	1.554	1.386
Perindopril	highest price medicinal product	2.242		1.777		1.638	1.461	Irbesartan	Lowest price medicinal product	1.228	0.824	0.737	0.658
Ramipril	Lowest price medicinal product	0.172		0.155		0.148	0.132	Irbesartan	highest price medicinal product	5.148	4.639	4.448	1.533
Ramipril	highest price medicinal product	1.061		0.956		0.916	0.786	Candesartan	Lowest price medicinal product	0.449	0.384	0.359	0.320
Quinapril	Lowest price medicinal product	0.987		0.890		0.853	0.761	Candesartan	highest price medicinal product	2.972	3.451	2.104	1.623
Quinapril	highest price medicinal product	1.988		1.791		1.188	1.060	Telmisartan	Lowest price medicinal product	0.941	0.770	0.710	0.634
Fosinopril	Lowest price medicinal product	0.991		0.893		1.258	1.121	Telmisartan	highest price medicinal product	2.334	2.103	1.836	1.638
Fosinopril	highest price medicinal product	2.169		1.954		1.874	1.652	olmesartan	Lowest price medicinal product	3.904	1.091	1.128	1.006
Trandolapril	Lowest price medicinal product	0.950		0.856		1.127	0.778	olmesartan	highest price medicinal product	8.070	8.955	6.709	4.024
Trandolapril	highest price medicinal product	3.172		2.859		2.741	2.444	Ca-antagonists					
Zofenopril	Lowest price medicinal product	3.568		2.923		2.744	2.487	amlodipine	Lowest price medicinal product	0.141	0.127	0.121	0.121
Zofenopril	highest price medicinal product	6.470		5.830		5.591	4.986	amlodipine	highest price medicinal product	5.925	5.340	5.120	0.922
B- blockers								felodipine	Lowest price medicinal product	0.393	0.354	0.339	0.339
Metoprolol	Lowest price medicinal product	1.169	1.091		1.015		1.015	felodipine	highest price medicinal product	2.591	2.335	1.977	2.972
Metoprolol	highest price medicinal product	5.447	6.977		6.691		6.691	nifedipine	Lowest price medicinal product	0.643	0.570	0.548	0.548
Atenolol	Lowest price medicinal product	0.637	0.574		0.550		0.550	nifedipine	highest price medicinal product	1.077	0.970	0.930	0.970
Atenolol	highest price medicinal product	0.913	0.823		0.577		0.577	nimodipine^*^		16.685	15.036	14.419	14.419
bisoprolol	Lowest price medicinal product	0.450	0.406		0.370		0.370	lacidipine^*^		1.508	1.360	1.304	1.304
Bisoprolol	highest price medicinal product	4.668	4.206		4.034		6.180	lercanidipine	Lowest price medicinal product	1.026	0.661	0.847	0.847
Nebivolol	Lowest price medicinal product	1.181	0.864		0.828		0.828	lercanidipine	highest price medicinal product	2.217	1.501	1.695	2.017
Nebivolol	highest price medicinal product	3.560	3.208		3.077		3.077	-					
Carvedilol	Lowest price medicinal product	1.106	0.997		0.956		0.956	-					
Carvedilol	highest price medicinal product	11.929	10.750		10.309		5.749	-					

^*^One trademark is available on the market.

## Conclusion

Patients with hypertension in Bulgaria have access to affordable first-line antihypertensive medicines. Despite the stable and low prices, the utilization mainly decreases. The reimbursed amount is reduced with a low rate or remains similar to that found at the beginning of the observed period. The results of the implemented budget cap as a measure to control NHIF cost are not evident and not fully expressed on the market for the first-line antihypertensive therapy.
